# The Research of Improved Grey GM (1, 1) Model to Predict the Postprandial Glucose in Type 2 Diabetes

**DOI:** 10.1155/2016/6837052

**Published:** 2016-05-23

**Authors:** Yannian Wang, Fenfen Wei, Changqing Sun, Quanzhong Li

**Affiliations:** ^1^School of Information Engineering, Zhengzhou University, Zhengzhou 450001, China; ^2^College of Public Health, Zhengzhou University, Zhengzhou 450001, China; ^3^Department of Endocrinology, Henan People's Hospital, Zhengzhou 450003, China

## Abstract

Diabetes may result in some complications and increase the risk of many serious health problems. The purpose of clinical treatment is to carefully manage the blood glucose concentration. If the blood glucose concentration is predicted, treatments can be taken in advance to reduce the harm to patients. For this purpose, an improved grey GM (1, 1) model is applied to predict blood glucose with a small amount of data, and especially in terms of improved smoothness it can get higher prediction accuracy. The original data of blood glucose of type 2 diabetes is acquired by CGMS. Then the prediction model is established. Finally, 50 cases of blood glucose from the Henan Province People's Hospital are predicted in 5, 10, 15, 20, 25, and 30 minutes, respectively, in advance to verify the prediction model. The prediction result of blood glucose is evaluated by the EGA, MSE, and MAE. Particularly, the prediction results of postprandial blood glucose are presented and analyzed. The result shows that the improved grey GM (1, 1) model has excellent performance in postprandial blood glucose prediction.

## 1. Introduction

As one of the major diseases that harm human health, diabetes can lead to many complications, including atherosclerosis, blindness, renal failure, and feet disorders with risk of amputation [1, 2]. According to the WHO, there are nearly 347 million diabetics in the world. The clinical presentation of diabetes includes hyperglycemia and hypoglycemia. And the main purpose of the clinical treatment of diabetes is to keep good control of blood glucose concentration. If the future blood glucose concentration is predicted, doctors and patients can take some action in advance to reduce the harm to patients. Compared with fasting glucose, postprandial blood glucose is more harmful because it tends to fluctuate. The Guideline for Management of Postprandial Blood Glucose also emphasizes that controlling of postprandial blood glucose has extremely profound significance. In order to reduce the risk of complications [3] and carefully control blood glucose levels in advance, effective postprandial blood glucose prediction method should be studied and it can provide support for doctors and patients.

CGMS (Continuous Glucose Monitoring System) is a device that is placed on the patient and used to measure patient's blood glucose every 5 minutes. Based on the blood glucose data provided by CGMS, many kinds of prediction methods of blood glucose were proposed, such as adaptive blood glucose prediction model [4], AR (autoregressive) model [5], neural network prediction model [6], and SVM (support vector machine) model [7]. Peng et al. [4] applied Kalman filter to smooth the blood glucose data from the CGMS, using AR model to build up the blood glucose prediction model, and the result showed that the blood glucose changes can be dynamically captured and the future blood glucose can be predicted. Wang and An [5] also adopted the AR model in predicting blood glucose; the result showed that the prediction was accurate with simple calculation, but their research did not take into account the smoothness of the original data. Tresp et al. [6] utilized neural network algorithm to predict blood glucose and found that their model had good tracking ability. Georga et al. [7] employed SVM algorithm to predict blood glucose and found that it had good prediction effectiveness when it had a large amount of data. In terms of selecting or training model parameters, if the original data sequence is longer, their prediction results are reliable, but if the original data sequence is shorter, due to inadequate information and lack of significant regularity, their forecasting accuracy is low.

The grey GM (1, 1) model not only has simple principle, less samples, easy calculation, high forecasting accuracy, and easy inspection but also can preprocess the original data, obtain better smoothness, and predict more effectively. In this paper, an improved grey GM (1, 1) model was proposed to make predictions in 30 min in advance on 72 hours of blood glucose and 2 hours of postprandial blood glucose, respectively; the experiment was performed on the MATLAB and the results were compared with AR model.

## 2. Materials and Methods

Grey GM (1, 1) model has been widely applied in many fields, such as economy, science, and education [8]. Grey GM (1, 1) model is a kind of homogeneous exponential growth model based on the accumulation generation sequence and the least squares method. The growth trend of original data has great influence on the accuracy of prediction. If the original data sequence is smooth, the more close to the exponential growth it is, the higher prediction precision the model can produce. The improved grey prediction model preprocesses the original data to improve the smoothness of the data sequence and greatly improve the prediction precision and the predictive value of original data sequence was obtained through an inverse transformation.

### 2.1. The Blood Glucose Prediction Model Based on the Improved Grey GM (1, 1) Model

Suppose an original data sequence *X* ^(0)^ is as the following formula:

(1) $$\begin{array}{c} X^{\left(0\right)}=\left(\;x^{\left(0\right)}\left(\;1\;\right),x^{\left(0\right)}\left(\;2\;\right),\ldots ,x^{\left(0\right)}\left(\;n\;\right)\;\right), \end{array}$$

where *x* ^(0)^(*i*) > 0, *i* = 1,2,…, *n*. The steps of establishing the blood glucose prediction model based on the data sequence *X* ^(0)^ are as follows.

(1) As a logarithmic transformation on *X* ^(0)^, mark *y* ^(0)^(*i*) = ln⁡*x* ^(0)^(*i*), *i* = 1,2,…, *n*. The data sequence *Y* ^(0)^ can be processed as the following formula:

(2) $$\begin{array}{c} Y^{\left(0\right)}=\left(\;y^{\left(0\right)}\left(\;1\;\right),y^{\left(0\right)}\left(\;2\;\right),\ldots ,y^{\left(0\right)}\left(\;n\;\right)\;\right). \end{array}$$

(2) Generate the accumulation generation sequence *Y* ^(1)^ by 1-AGO, as in the formula

(3) $$\begin{array}{c} Y^{\left(1\right)}=\left(\;y^{\left(1\right)}\left(\;1\;\right),y^{\left(1\right)}\left(\;2\;\right),\ldots ,y^{\left(1\right)}\left(\;n\;\right)\;\right), \end{array}$$

where *y* ^(1)^(1) = *y* ^(0)^(1), *y* ^(1)^(*k*) = ∑_*i*=1_^*k*^ *y* ^(0)^(*i*) (*k* = 2,3,…, *n*).

(3) Through the first-order accumulative generation sequence *Y* ^(1)^, GM (1, 1) model is established; a first-order differential equation can be gotten as the formula

(4) $$\begin{array}{c} \frac{dy^{\left(1\right)}t}{dt}+ay^{\left(1\right)}\left(\;t\;\right)=b, \end{array}$$

where *a* is the development coefficient and *b* is the control variable. Get the corresponding form of grey differential equation as the following formula:

(5) $$\begin{array}{c} x^{\left(0\right)}\left(\;i\;\right)+az^{\left(1\right)}\left(\;i\;\right)=b,\;i=2,3,\ldots . \end{array}$$

(4) To solve the parameters *a* and *b*, parameters *∅* = [*a*, *b*]^*T*^ can be determined by the least square method as the formula

(6) $$\begin{array}{c} \emptyset ={\left[\;B^{T}B\;\right]}^{-1}B^{T}Y, \end{array}$$

where

(7) B=−z121−z131⋮⋮−z1n1,z1k=12y1i+y1i−1,Y=x02,x03,…,x0nT.

(5) The known initial condition is ${\hat{y}}^{\left(1\right)}(1)=y^{\left(1\right)}\left(1\right)=y^{\left(0\right)}\left(1\right)$; put it into formula (6) and formula (5) to obtain the generated data sequence ${\hat{y}}^{\left(1\right)}$ as the following formula:

(8) y^1i=y01−b^a^e−a^i−1+b^a^i=2,3,…,n.

(6) The known initial condition is ${\hat{y}}^{\left(1\right)}(1)=y^{\left(1\right)}\left(1\right)=y^{\left(0\right)}\left(1\right)$,

(9) $$\begin{array}{c} {\hat{y}}^{\left(0\right)}\left(\;i\;\right)={\hat{y}}^{\left(1\right)}\left(\;i\;\right)-{\hat{y}}^{\left(1\right)}\left(\;i-1\;\right),\;i=2,3,\ldots ,n. \end{array}$$

Put (8) into (9) and get the data sequence ${\hat{y}}^{\left(0\right)}$ as the formula

(10) y^0i=1−ea^x01−b^a^e−a^i−1,i=2,3,…,n.

(7) The known inverse transformation is as formula

(11) $$\begin{array}{c} {\hat{x}}^{\left(0\right)}\left(\;i\;\right)=e^{{\hat{y}}^{\left(0\right)}\left(i\right)}. \end{array}$$

Put (10) into (11) and get the forecast sequence of the original blood data sequence ${\hat{x}}^{\left(0\right)}$ as the formula

(12) $$\begin{array}{c} {\hat{x}}^{\left(0\right)}\left(\;i\;\right)=e^{\left(1-e^{\hat{a}}\right)\left(x^{\left(0\right)}\left(1\right)-\hat{b}/\hat{a}\right)e^{-\hat{a}\left(i-1\right)}},\;i=2,3,\ldots ,n. \end{array}$$

Put *i* = 2,3,…, *n* into formula (12), the fitted values of original data can be gotten. When *i* > *n*, the predictive values of blood glucose can be gotten.

### 2.2. The Ways of Testing the Accuracy of the GM (1, 1) Model

The improved GM (1, 1) model must be strictly examined and meet some requirements before being predicted. Three ways were used to estimate the accuracy of the GM (1, 1) model: relative error size test, posterior deviation test, and correlation test.

*(1) The Relative Error Size Test*. The relative error size test is a kind of arithmetic test by intuitively comparing the data point by point, which observes whether the relative error meets the requirement by comparing the prediction data and the actual data of blood glucose. The relative error of GM (1, 1) model is *ε*(*i*) as formula

(13) $$\begin{array}{c} \varepsilon \left(\;i\;\right)=\frac{e\left(i\right)}{x^{\left(0\right)}\left(i\right)}\times 100\%=\frac{x\left(i\right)-\hat{x}\left(i\right)}{x^{\left(0\right)}\left(i\right)}\times 100\%, \end{array}$$

where $\varepsilon \left(i\right)=x^{\left(0\right)}\left(i\right)-{\hat{x}}^{\left(0\right)}\left(i\right)$, *i* = 1,2,…, *n*. The average relative error of GM (1, 1) model is $\overset{-}{\varepsilon }$ as the following formula:

(14) $$\begin{array}{c} \overset{-}{\varepsilon }=\frac{1}{n}\overset{n}{\underset{i=1}{\sum }}\left|\;\varepsilon \left(\;i\;\right)\;\right|. \end{array}$$

The accuracy of GM (1, 1) model is *p*° as the formula

(15) $$\begin{array}{c} p^{{}^{\circ}}=\left(\;1-\overset{-}{\varepsilon }\;\right)\times 100\%. \end{array}$$

General requirement is *p*° > 80%.

*(2) The Posterior Deviation Test*. The posterior deviation test is of statistical method and makes inspection according to the probability distribution of residual. The indexes of the posterior variance ratio *C* and the posterior probability *p* are the two key indicators. The *C* should be as small as possible and *p* should be as large as possible. According to the size of the *C* and *p*, the precision of the model can be divided into four levels “superior, qualified, marginal, and disqualified” [9]. The level of model accuracy is max⁡{*p*, *C*}.

Calculate the a posteriori variance ratio *C* as formula

(16) $$\begin{array}{c} C=\frac{S_{2}}{S_{1}}, \end{array}$$

where $S_{1}^{2}=(1/n)\sum_{i=1}^{n}{\left(x^{\left(0\right)}\left(i\right)-{\overset{-}{x}}^{\left(0\right)}\right)}^{2}$ and $S_{2}^{2}=(1/n)\sum_{i=1}^{n}{\left(e(i)-\overset{-}{e}\right)}^{2}$.

Calculate the posterior probability *p* as formula

(17) $$\begin{array}{c} p=p\left\{\;\left|\;e\left(\;i\;\right)-\overset{-}{e}\;\right|<0.6745S_{1}\;\right\}, \end{array}$$

where $\overset{-}{e}=(1/n)\sum_{i=1}^{n}e(i)$.

*(3) The Correlation Test*. The correlation test is a kind of geometry inspection, used to investigate similarity of the predictive value curve and the actual value curve. In general, the closer the geometry is, the closer the change trend is and the greater the correlation is.

The correlation degree of the GM (1, 1) model is *ξ* as the formula

(18) $$\begin{array}{c} \xi =\frac{1}{n}\overset{n}{\underset{i=1}{\sum }}\xi_{i}, \end{array}$$

where *ξ*_*i*_ is the correlation coefficient of ${\hat{X}}^{\left(0\right)}$ and *X* ^(0)^ as the following formula:

(19) ξi=min1≤i≤n⁡x0i−x^0i+ρ max1≤i≤n⁡x0i−x^0ix0i−x^0i+ρ max1≤i≤n⁡x0i−x^0i,

where *ρ* ∈ [0,1] is the resolution ratio, *ρ* = 0.5 [10].

General requirement is *ξ* > 0.6 [10], and the greater the correlation is, the better the prediction is.

### 2.3. Model Evaluation Indicators

In order to evaluate forecast results, the present study applied three performance indicators: EGA (Clark Error Grid Analysis), MSE (Mean Square Error), and MAE (Mean Absolute Error).

The EGA [11, 12] is divided into A, B, C, D, and E regions. In region A, the prediction effect is the best, while in region E it is poor. The EGA is used to evaluate the accuracy and the precision of prediction method of blood glucose and provide guidance to doctors and patients. So far, the EGA has been accepted as one of the “gold standards” in evaluating the accuracy of predicting blood glucose.

The MSE is as the formula

(20) $$\begin{array}{c} MSE=\sqrt{\frac{\sum {\left(e\left(i\right)\right)}^{2}}{n}},\;i=1,\ldots ,n. \end{array}$$

The MAE is as the formula

(21) $$\begin{array}{c} MAE=\frac{\sum \left|e\left(i\right)\right|}{n},\;i=1,\ldots ,n. \end{array}$$

## 3. Results

### 3.1. The Data

**Figure 1:**
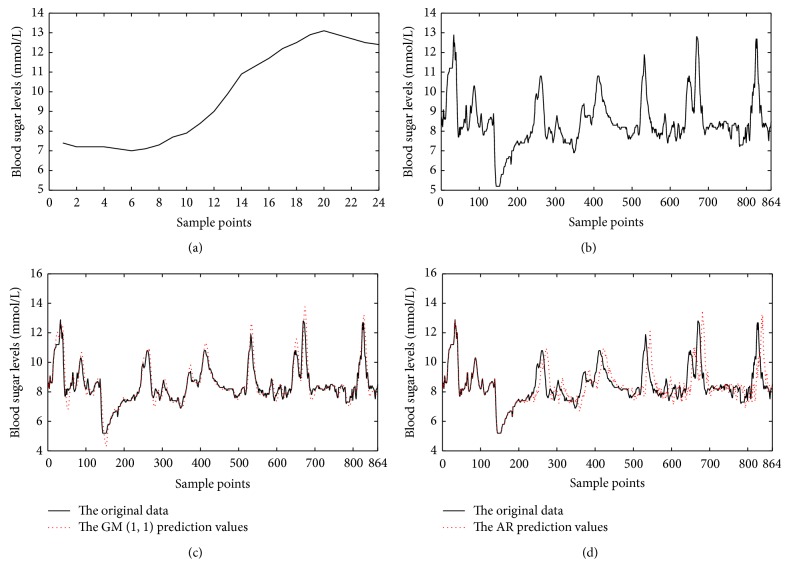
(a) Patient A: blood glucose levels of 2 hours after meal. The front 18 samples constituted the original blood glucose sequence. The following 6 samples are the prediction sequence. (b) Patient A: blood glucose levels of 72 hours. The front 200 samples constituted the original glucose sequence. The following 664 samples are the prediction sequence. (c) The prediction results of (b). The original blood glucose data are shown by the black curve. The improved GM (1, 1) prediction values are shown by the red curve. (d) The prediction results of (b). The original blood glucose data are shown by the black curve. The AR model prediction values are shown by the red curve.

The data used in this study is obtained from the Henan Provincial People's Hospital, where nurses used CGMS to acquire the blood glucose in patients as original data sequence of experiment. CGMS receives a current signal every 10 seconds and averages and converts it into blood glucose values every 5 minutes. The CGMS can continuously work at least 72 hours and store 864 blood glucose values. One of the patients' blood glucose concentration of 2 hours after meal is shown in Figure 1(a), and one of the patients' blood glucose concentration of 72 hours is shown in Figure 1(b). 50 cases of blood glucose will be employed to verify the prediction model based on the GM (1, 1) model and compared with the AR model.

### 3.2. Prediction Result of Figure 1(b)

**Figure 2:**
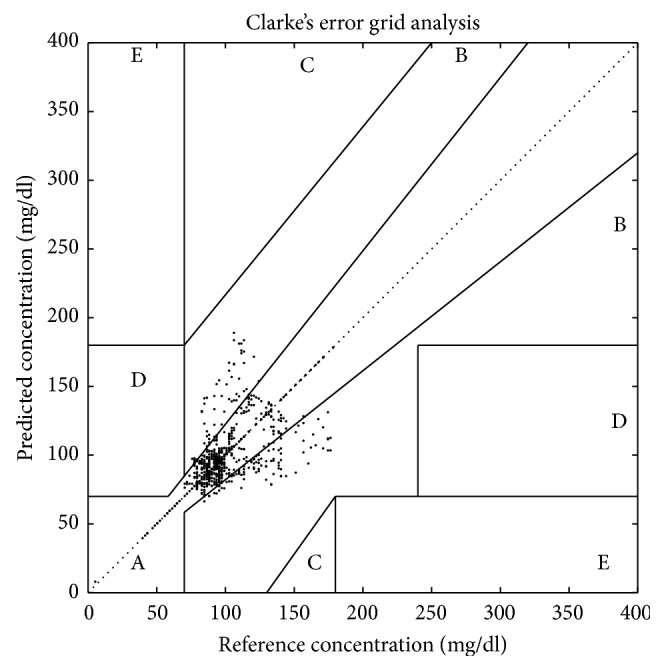
Clark Error Grid Analysis result. The result is calculated according to the original blood glucose data and the improved GM (1, 1) prediction values in Figure 1(c). In this figure, the 91.72% of the error points lie in zone A and the remaining 8.22% in zone B.

The front 200 samples' data of Figure 1(b) is employed to establish the GM (1, 1) model for forecasting the following 664 blood glucose levels. Firstly, put the 200 samples (*n* = 200) into formula (1) as original data sequence *X* ^(0)^. Secondly, calculate as above the methods of the blood glucose prediction model. Then, test the accuracy of the model and do experiment which was conducted based on MATLAB. Finally, show the continuous prediction results in Figure 1(c). The prediction results of AR model are shown in Figure 1(d). By calculating, the result of MSE of Figure 1(c) is 1.0306 and MAE is 0.3307. The result of MSE of Figure 1(d) is 1.4356 and MAE is 0.5379. The results of EGA of Figure 1(c) are shown in Figure 2, and it is clear that the majority of the error points (91.72%) lie in zone A and the remaining 8.22% in zone B.

### 3.3. Prediction Result of Figure 1(a)

The front 18 samples data of Figure 1(a) are employed to establish the GM (1, 1) model for forecasting the following 6 blood glucose levels. Firstly, put the 18 samples (*n* = 18) into formula (1) as original data sequence *X* ^(0)^. Secondly, by calculating as above the methods of blood glucose prediction model, we rewrote formula (12) of blood glucose prediction model as the following formula:

(22) $$\begin{array}{c} {\hat{x}}^{\left(0\right)}\left(\;i\;\right)=90.701e^{0.02\left(i-1\right)}-88.7\;\left(i=2,3,\ldots ,18\right). \end{array}$$

Then, to test the accuracy of formula (22), experiment was conducted based on MATLAB. The results are *p*° = 97.5%, *C* = 0.17, *p* = 1, and *ξ* = 0.92.

Finally, formula (22) of prediction model based on GM (1, 1) fulfilled the requirements; the prediction values and the errors of blood glucose are shown in Table 1. That is, ${\hat{x}}^{\left(0\right)}\left(19\right)=11.9$, ${\hat{x}}^{\left(0\right)}\left(20\right)=12.4$, ${\hat{x}}^{\left(0\right)}\left(21\right)=12.9$, ${\hat{x}}^{\left(0\right)}\left(22\right)=13.4$, ${\hat{x}}^{\left(0\right)}\left(23\right)=14.0$, and ${\hat{x}}^{\left(0\right)}\left(24\right)=14.5$.

As shown in Figure 2, the improved grey GM (1, 1) model can be used for predicting exactly blood glucose. According to Table 1, the postprandial blood glucose prediction result of patient A, whether in MSE or MAE, is more ideal than the continuous 72 hrs predictions.

### 3.4. The Prediction Result of 50 Patients

We repeated experiments with 50 cases' data of the blood glucoses like patient A. The statistical errors of 50 cases are shown in Table 2.

As shown in Table 2, an improved grey GM (1, 1) model is applied to predict blood glucose with a small amount of data, and in particular in terms of improved smoothness it can get higher prediction accuracy than AR model, so is the prediction result of the 2 hrs after meal. This means that the improved grey GM (1, 1) model performs well not only 72 hrs of continuous prediction but also 2 hrs after the meal. In order to obtain more accurate prediction of postprandial blood glucose and provide support to doctors and patients, the improved grey GM (1, 1) model is simpler and more reliable in comparison with the AR prediction model and it only needs the historical data provided by CGMS [13, 14].

## 4. Discussion

In this paper, the improved grey prediction model in terms of smoothness is used to predict blood glucose of type 2 diabetes patients and the blood glucose for nearly 72 hrs and 2 hrs after meal, respectively, is predicted. The prediction result was compared with AR model. The results showed that the improved grey GM (1, 1) model has outperformed the AR model in predicting blood glucose, especially 2 hrs after meal.

In order to value the error in every stage clearly, a series of experiments was performed at three stages: 3 hrs after meal, 4 hrs after meal, and 6 hrs at night. The statistical errors in three stages of 50 cases are shown in Table 3. The error of 6 hrs at night is bigger than the error of 4 hrs after meal, and the error of 4 hrs after meal is bigger than the error of 3 hrs after meal. Additionally, as shown in Table 3, the error of 3 hrs after meal is bigger than the error of 2 hrs after meal. But the difference between the error of 6 hrs at night and the error of 2 hrs after meal is obviously bigger than the difference between the error of other stages and the error of 2 hrs after meal. This means that the prediction result of the improved grey GM (1, 1) model is worse than the AR model in the stage of 6 hrs at night. That is, the GM (1, 1) model has a more accurate predication result for the original sequences with wide fluctuation range than the ones with small fluctuation range. This disadvantage will be improved in the future research. Compared with the linear AR model, the GM (1, 1) model is better especially when the blood glucose levels suddenly rise or drop. Because the grey GM (1, 1) model is a kind of homogeneous exponential growth model, its predicting values may show the error of exponential at some sample points, but it can be resolved by correcting predicting value using real value in the GM (1, 1) model.

In a word, the method for predicting postprandial blood glucose based on the improved gray prediction model can be developed to help doctors and patients and in the future may help to develop an artificial pancreas, which adapts to the future changing of patient's blood glucose levels and gives better insulin outcomes as a result.

## Figures and Tables

**Table 1:** The prediction results of Figure 1(a).

Sample points	The real value	The prediction value	MSE	MAE
19	12.9	11.9	0.9786	0.1631
20	13.1	12.4		
21	12.9	12.9		
22	12.7	13.4		
23	12.5	14.0		
24	12.4	14.5		

MSE: mean square error; MAE: mean absolute error; the real value (mmol/L); the prediction value (mmol/L).

**Table 2:** The statistical errors of 50 cases.

	2 hrs after meal error		72 hrs error	
	MSE	MAE	MSE	MAE
AR model	1.8294	0.6549	5.6410	0.5211
GM (1, 1) model	0.9786	0.1631	5.4847	0.5145

MSE: mean square error; MAE: mean absolute error.

**Table 3:** The statistical errors in stages of 50 cases.

	3 hrs after meal error		4 hrs after meal error		6 hrs at night error	
	MSE	MAE	MSE	MAE	MSE	MAE
AR model	2.7709	1.4644	3.6996	1.4543	3.1302	1.1134
GM (1, 1) model	1.1558	0.7697	2.149	0.8335	5.2131	2.5436

MSE: mean square error; MAE: mean absolute error.
